# Exploring the antimicrobial, antiviral, antioxidant, and antitumor potentials of marine *Streptomyces tunisiensis* W4MT573222 pigment isolated from Abu-Qir sediments, Egypt

**DOI:** 10.1186/s12934-023-02106-1

**Published:** 2023-05-05

**Authors:** Walaa M. Ibrahim, Zakia A. Olama, G. M. Abou-elela, Heba S. Ramadan, Ghada E. Hegazy, Dalia El S. El Badan

**Affiliations:** 1 Botany & Microbiology Department, Faculty of Science, Alexandria University, Baghdad St, Moharam Bek, Alexandria, 21568 Egypt; 2 National Institute of Oceanography & Fisheries, NIOF-Egypt, Alexandria, Egypt; 3 Bioprocess Development Department, Genetic Engineering & Biotechnology Research Institute (GEBRI), City of Scientific Research & Technological Applications, Alexandria, Egypt; 4 Department of Biological Sciences, Faculty of Science, Beirut Arab University, Beirut, Lebanon

**Keywords:** Marine actinomycetes, Secondary metabolites, Pigments, Antibacterial

## Abstract

Due to the therapeutic importance of microbial pigments, these pigments are receiving the attention of researchers. In this present study 60 isolates were isolated from sediments of Abu-Qir coast of the Mediterranean sea, Alexandria, Egypt, out of which 12 were considered as pigmented actinomycetes. *Streptomyces* sp*.* W4 was characterized by small round green pigmented colonies when grown on starch-casein agar medium. The green pigment was extracted using a mixture of acetone-methanol (7:3 v/v). The antimicrobial, antioxidant, antiviral, and anticancer activities of the green pigment produced by *Streptomyces* sp.W4 were investigated. The pigment was characterized using FTIR, Raman spectroscopy, EDX and GC–MS. The results revealed that the pigment has antibacterial and antifungal activity and also showed inhibition of HAV 78% but its antiviral activity against the Adenovirus was weak. The results proved the safety of the pigment toward normal cells and anticancer activity against three different cancer cell lines HepG-2 (liver cancer cell line), A549 (lung cancer cell line), and PAN1 (pancreas cancer cell line). The pigment was combined with 9 antibiotics and then tested against the Gram-negative bacterium *Enterococcus faecalis* using disc diffusion bioassay. LEV showed an antagonistic effect, while CXM and CIP showed a synergistic effect.

## Introduction

Actinomycetes are a diverse group of Gram-positive filamentous bacteria. They belong to the Actinomycetales order, Phylum: Actinobacteria, Class: Actinobacteria, Subclass: Actinobacteridae [1, 2]. They are free-living, saprophytic bacteria, a major source of antibiotic development [3], widely spread in both natural and manmade environments, and play an important role in organic matter degradation [4]. They are the most economically and biotechnologically important prokaryotes capable of developing a wide variety of bioactive secondary metabolites, such as antibiotics, antitumor agents, immunosuppressive agents, and enzymes [1, 5] and also regarded as a rich source of antibiotics and bioactive molecules [1, 4]. Approximately 23 000 bioactive secondary metabolites were identified by microorganisms, and actinomycetes produce over 10,000 of these compounds. The importance of actinomycetes to society in providing useful drugs, especially antibiotics and anticancer agents, and to the pharmaceutical industry for revenue-generating a discovery platform, is unquestionable [6, 7]. Pigments are generally classified into natural pigments and synthetic pigments. Natural pigments of microbial origin come from bacteria, fungi, and actinomycetes. Some of the most important natural pigments are carotenoids, flavonoids, tetrapyrroles, and some xanthophylls such as astaxanthin, melanin, voilacein, and riboflavin [8]. Parmar et al. [9] reported that actinomycetes are biologically important microorganisms and well known for their importance for human welfare. Actinomycetes are capable of producing a wide range of bioactive compounds, as well as natural pigments. The Frequency of resistance in microbial pathogens continues to grow at an alarming rate throughout the world [10]. To overcome this problem, the production of newer, more powerful drugs without any side effects is critical. In general, the antibacterial activity of marine actinobacteria has been extensively studied. The characterization of several antifungal peptides (AFPs) produced by actinomycetes and their biological activities against plant diseases were discussed by [11]. Several reports have shown promising antioxidant activity of *Streptomyces* species isolated from soil or ocean. Marine habitats provide more chances for isolating new species of *Streptomyces* with unique chemical structures repelling many microbial diseases and cancers [6, 12]. Thenmozhi and Kannabiran [13] reported that marine actinomycetes are potential providers of new bioactive metabolites. The main findings of Berezin et al. [14] showed that extracts from actinomycetes showed antiviral activity against influenza viruses, Sendai virus, and Newcastle Disease Virus. Cancer remains one of the most serious human health problems and breast cancer is the second most universal cause of cancer deaths in women [15]. Many of the antitumor compounds that are isolated from marine drugs are derived from marine actinobacteria and these metabolites play an important role in the identification of pharmaceutical compounds [16]. The objective of this work is to study the antimicrobial, antiviral, antioxidant, and antitumor potentials of marine *Streptomyces tunisiensis* W4MT573222 pigment for medical purposes.

## Results

### Isolation and screening

In this study, ten sediment samples were collected under aseptic conditions from different locations on the Abu-Qir coast of the Mediterranean Sea in Egypt and were stored at 4 °C until use.

60 actinomycete isolates were isolated, out of which (12) were considered pigmented actinomycetes using different cultivation approaches. All isolates were screened for the production of some antimicrobial agents. Unique characteristics of the isolate which was coded W4 in comparison to other isolates were observed as it was able to produce obvious and unique green pigment therefore it was selected for the completion of this study.

### Phenotypic and molecular characterization of the selected isolate W4

**Fig. 1:**
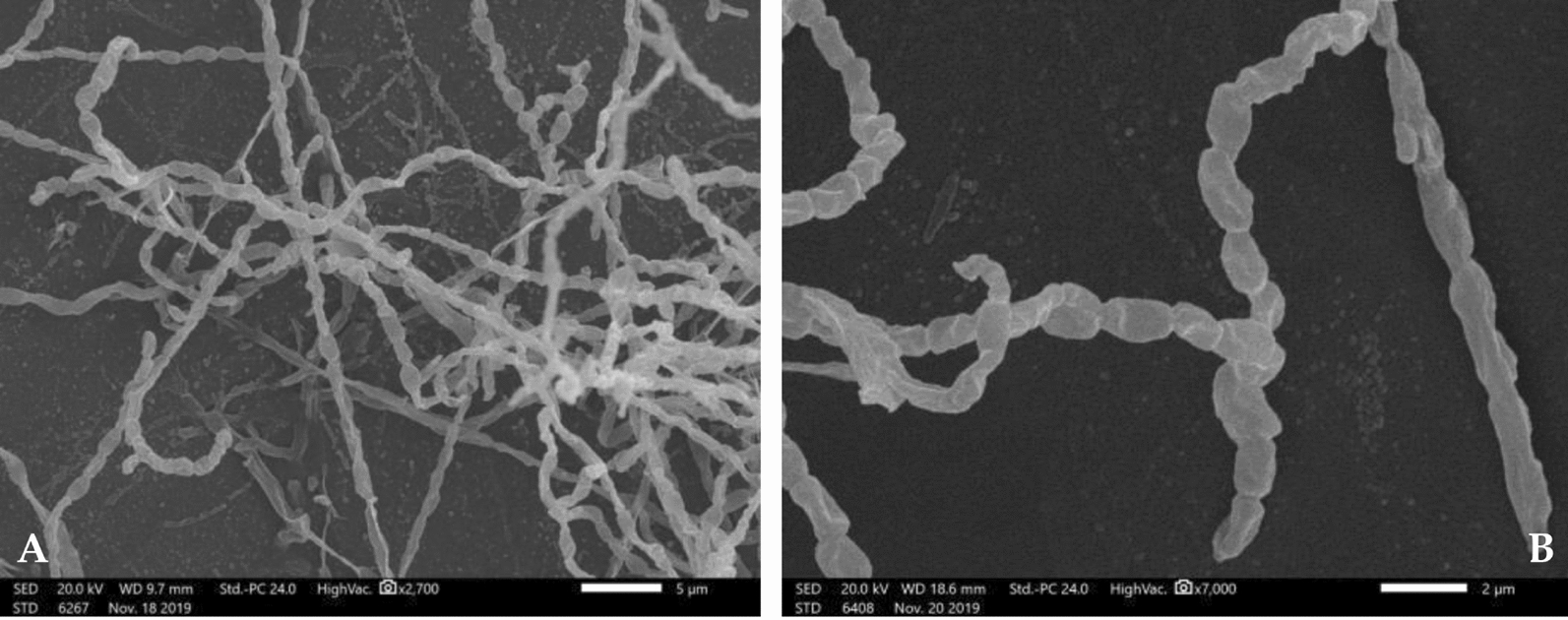
Scanning electron micrograph of the selected isolate W4 showing branching mycelia (**A**), mycelia with spores (**B**)

**Fig. 2:**
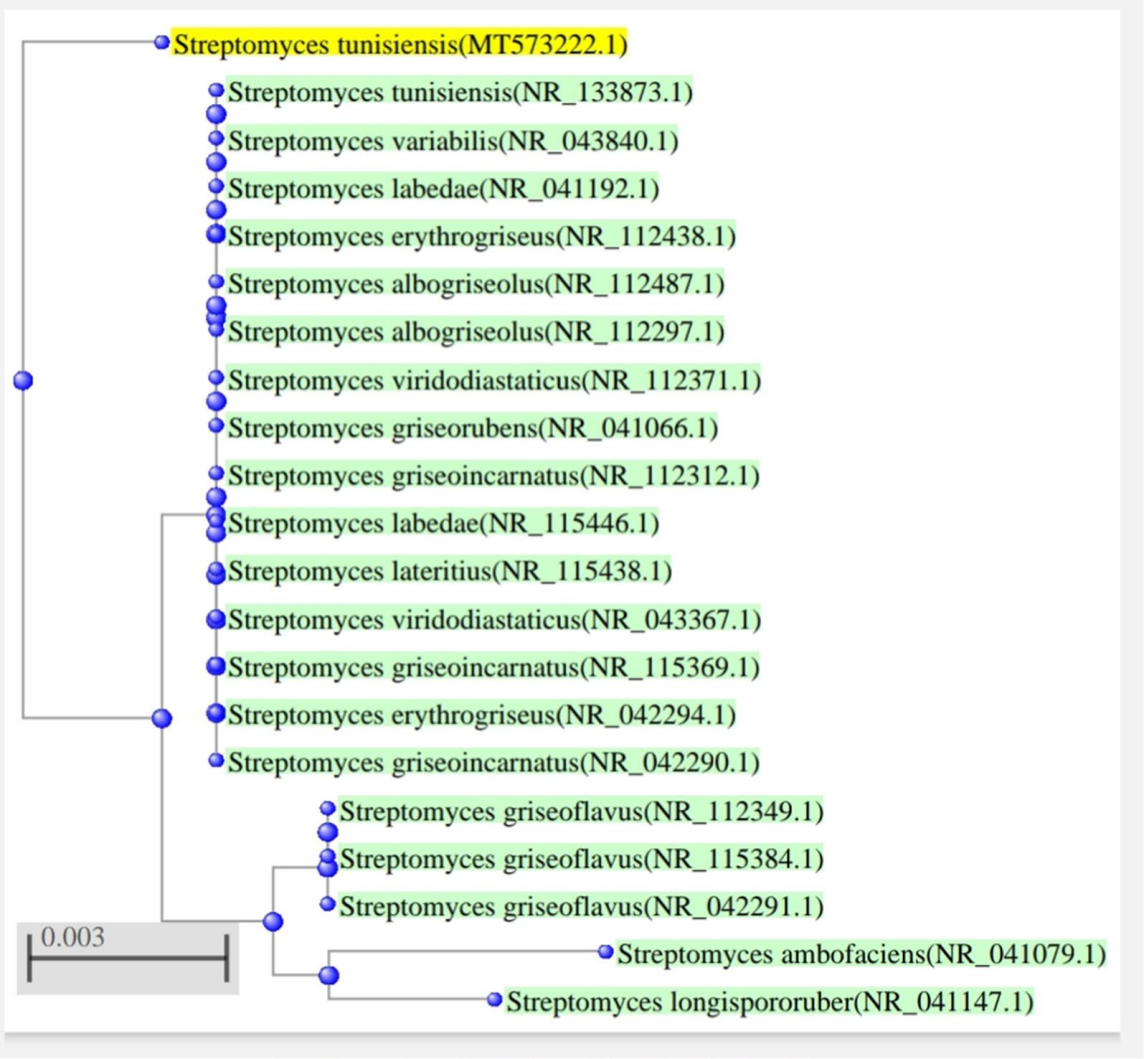
Phylogenetic tree, based on the 16S rRNA gene sequence, isolate W4 and the closest relatives. The tree was obtained by the neighbor-joining method

Isolate W4 is Gram-positive and forms small, round, smooth, green-pigmented colonies after one week of incubation at 35 ± 2 °C. Scanning electron microscopy (SEM) examination revealed the formation of branching mycelia (Fig. 1A) which contained spores (Fig. 1B). The genomic DNA of isolate W4 was extracted and the gene coding for 16SrRNA was partially amplified using the universal primers. The product of the PCR was analyzed on 1% agarose gel stained with ethidium bromide. Finally, isolateW4 was identified by sequencing the PCR-amplified 16S rRNA gene with 1500 bp, then the BLAST program was used to assess the similarity. The 16S rRNA of isolate W4 was submitted to GeneBank under accession number (MT573222). The isolate was identified as *Streptomyces tunisiensis* W4MT573222, Fig. 2**.**

### Identification of the extracted pigment

The fermentation of *Streptomyces tunisiensis* W4MT573222 was carried out according to our optimized conditions. Optimization of the pigment production from *Streptomyces tunisiensis* W4MT573222 has been carried out using Plackett–Burman and Box-Behnken experimental designs. According to Plackett–Burman and Box-Behnken experimental results, an optimum response for pigment production is predicted with the following medium composition (g/L): starch, 5 g; KNO_3_, 0.88 g; K_2_HPO_4_, 1 g; MgSO_4_, 0.025 g; FeSO_4_, 0.015 g; Casein, 0.65 g; and CaCO_3_, 0.03 g. The results indicate that the optimized conditions accelerated the pigment production and the produced pigment was about 12.2 fold increase than that recorded with the basal condition. By incubation at 35 ± 2 °C with shaking at 120 rpm for 7 days and with an inoculum age7 days. Pigments were then extracted by liquid extraction using acetone and methanol (7:3v/v). UV, Fourier transform infrared (FT-IR), Raman spectroscopy, EDX, and GC–MS were carried out to elucidate the chemical structure of the partially purified pigment.

**Fig. 3:**
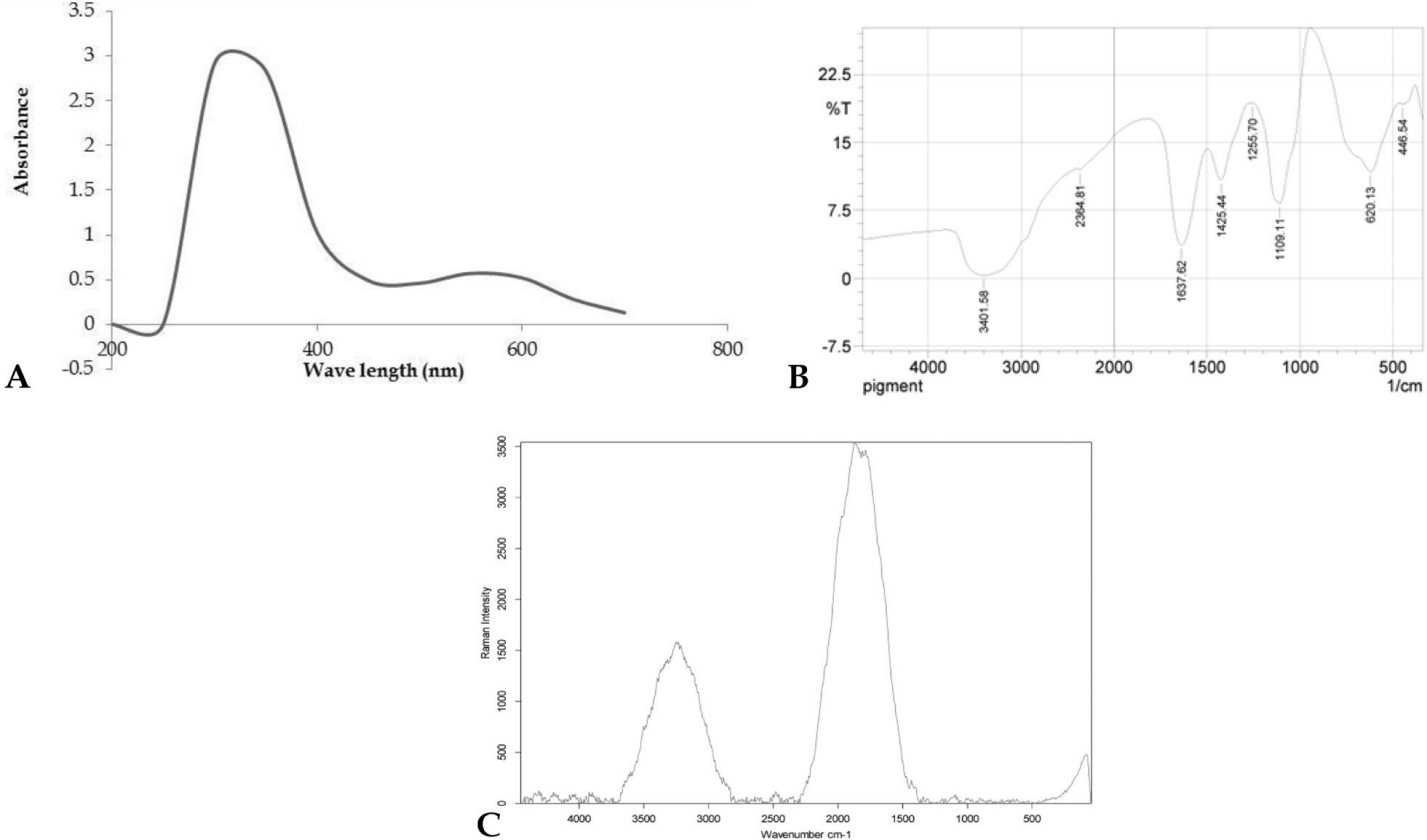
UV–Visible spectra (**A**), Fourier-Transform Infrared spectrum (**B**) and Raman spectra (**C**) of the tested pigment

UV–Vis spectrum: the wavelength at which the pigment has its strongest photon absorption was at 300 nm (λ_max_ = 300 nm) as shown in (Fig. 3A).

The presence of several chemical groups in the green pigment was discovered by FTIR spectroscopic analysis, as demonstrated in (Fig. 3B). The peaks at 446.54 and 620.13 cm^−1^ correspond to the stretching frequencies of C–I and C–Br (alkyl halides), respectively. C–O and C–O–C are represented by bands at 1109.11 cm^−1^ and 1255.70 cm-1 respectively. The peak at 1425.44 cm^−1^ corresponded to O–H_._ The strong peak at 1637.62 cm^−1^ is assigned to the C=C alkene stretching which means that some aliphatic compounds have existed in the green pigment. The peak at 2364.81 cm^−1^ is assigned to C-N and the peak at 3401.58 cm^−1^ is assigned to N–H which means some amine functional groups have existed in the green pigment.

The Raman spectroscopy analysis (Fig. 3C) showed the presence of three peaks with two strong and one weak intensity of the green pigment, with a strong band between 1800 and 2000 cm^−1^ which corresponds to C=O bond, and another less strong band between 3200 and 3650 cm^−1^ corresponds to O–H bond.

The EDX clarified the presence of carbon, oxygen, magnesium, sulfur, sodium and chlorine elements in the pigment structure. It has been found that Oxygen, Magnesium, sodium, and chlorine were the key elements in the pigment structure.

**Fig. 4:**
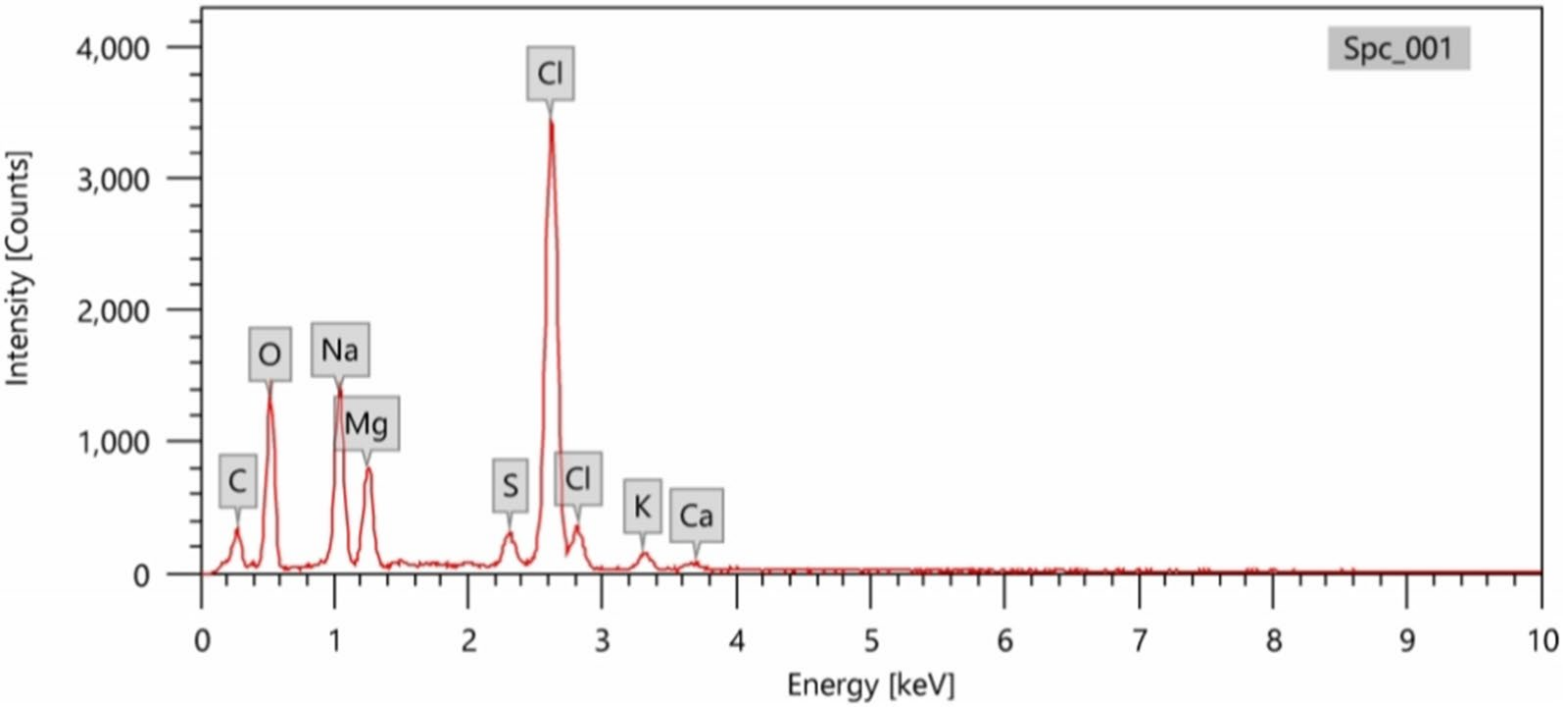
Energy Dispersive X-ray analysis (EDX) of *Streptomyces tunisiensis*W4MT573222 pigment

The elemental map of the extracted pigment produced by *Streptomyces tunisiensis* W4MT573222 was demonstrated by EDX analysis as shown in Fig. 4 The two absorption peaks corresponding to Cl, Na, and O are most abundant in the sample, while the minor amount of K and Ca were detected.

### GC–MS analysis

**Fig. 5:**
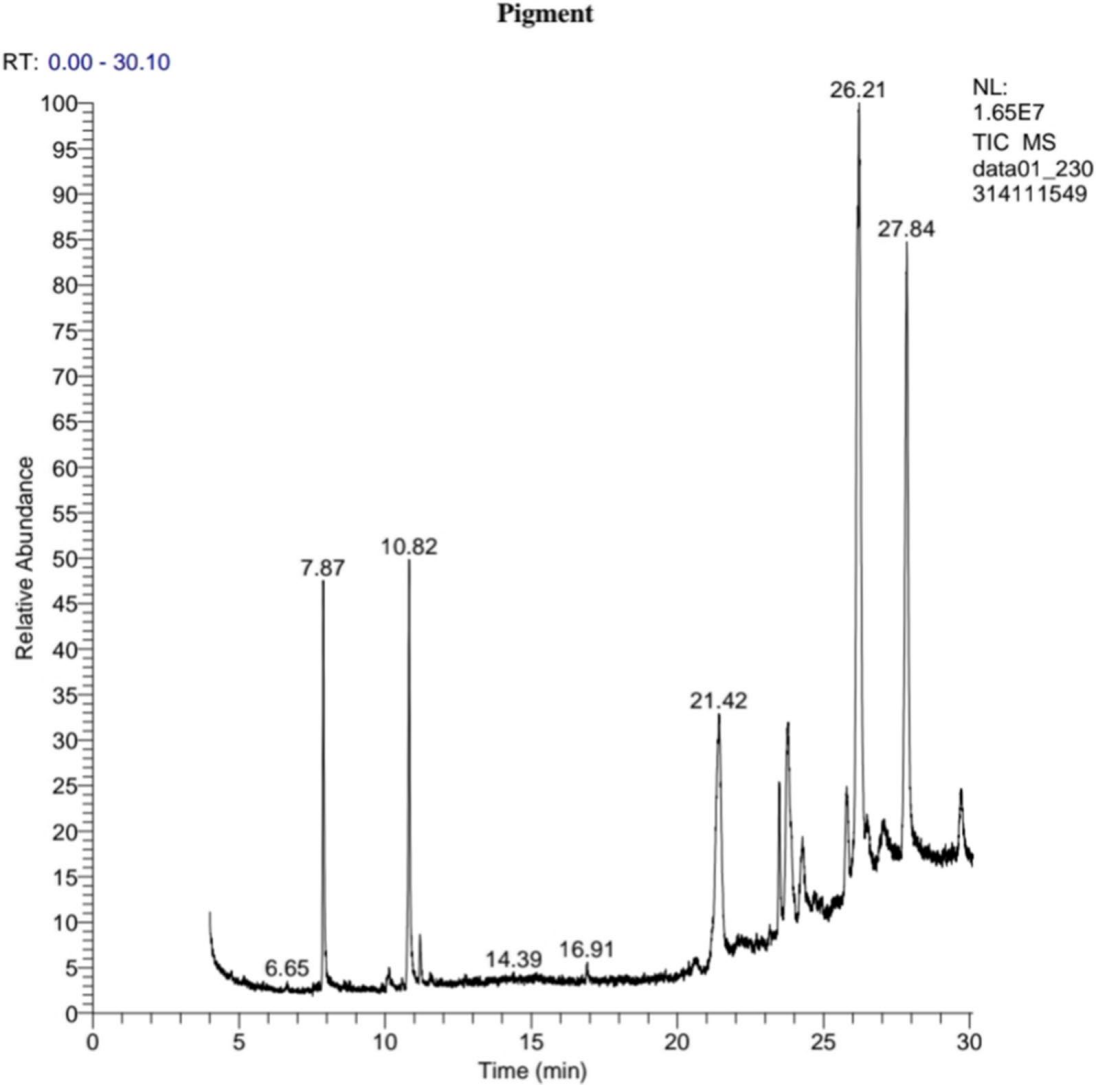
GC–MS chart analysis of* Streptomyces tunisiensis* W4MT573222 pigment

GC–MS analysis for the partially extracted pigment shown in Fig. 5 indicated the presence of different components. The gas chromatogram showed the relative concentrations of various compounds getting eluted as a function of retention time. Seven major compounds have been detected from GC–MS analysis for the extracted pigment produced by *Streptomyces tunisiensis* W4MT573222, including Hexadecanoic acid, methyl ester., 9-Octadecenoic acid (Z), methyl ester., 2-pentyl-Thiophene., 3-[1-[p-Chlorophenyl]-1-ethoxyethyl]piperidine., Cyclohexane, 1-bromo-3-methyl., Dodecane,1-cyclopentyl-4-(3-cyclopentylpropyl)., and Octadecanoic acid, 2-propenylester at a retention time of 7.87, 10.82, 21.42, 24.69, 26.21, 27.84, and 29.70 min respectively. The most abundant peak was obtained by Dodecane,1-cyclopentyl-4-(3-cyclopentylpropyl) (19.20%) followed by 2-pentyl-Thiophene (11.83%) with retention time 27.84 and 21.42 min respectively, and the least abundant peak was obtained by 3-[1-[p-Chlorophenyl]-1-ethoxyethyl]piperidine (0.30%) with 24.69 min retention time. The molecular formula for the seven identified components in extracted pigment produced by *Streptomyces tunisiensis* W4MT573222 are shown in Table 1.

**Table 1:** Chemical constituents of methanolic extract of pigment produced by *Streptomyces tunisiensis* W4MT573222 through GC–MS

RT (min)	Probability	Compound name	Area%	Molecular formula	Molecular weight
7.87	40.90	Hexadecanoic acid, methyl ester	5.67	C_17_H_34_O_2_	270
10.82	14.36	9-Octadecenoic acid (Z), methyl ester	6.77	C_19_H_36_O_2_	296
21.42	20.24	2-pentyl-Thiophene	11.83	C_9_H_14_S	154
24.69	5.59	3-[1-[p-Chlorophenyl]-1-ethoxyethyl]piperidine	0.30	C_15_H_22_ClNO	267
26.21	17.69	Cyclohexane, 1-bromo-3-methyl	33.03	C7H13Br	176
27.84	9.63	Dodecane,1-cyclopentyl-4-(3-cyclopentylpropyl)	19.20	C_25_H_48_	348
29.70	13.31	Octadecanoic acid, 2-propenylester	1.92	C_21_H_40_O_2_	324

### Antibacterial activity of *Streptomyces tunisiensis*W4MT573222 pigment

**Fig. 6:**
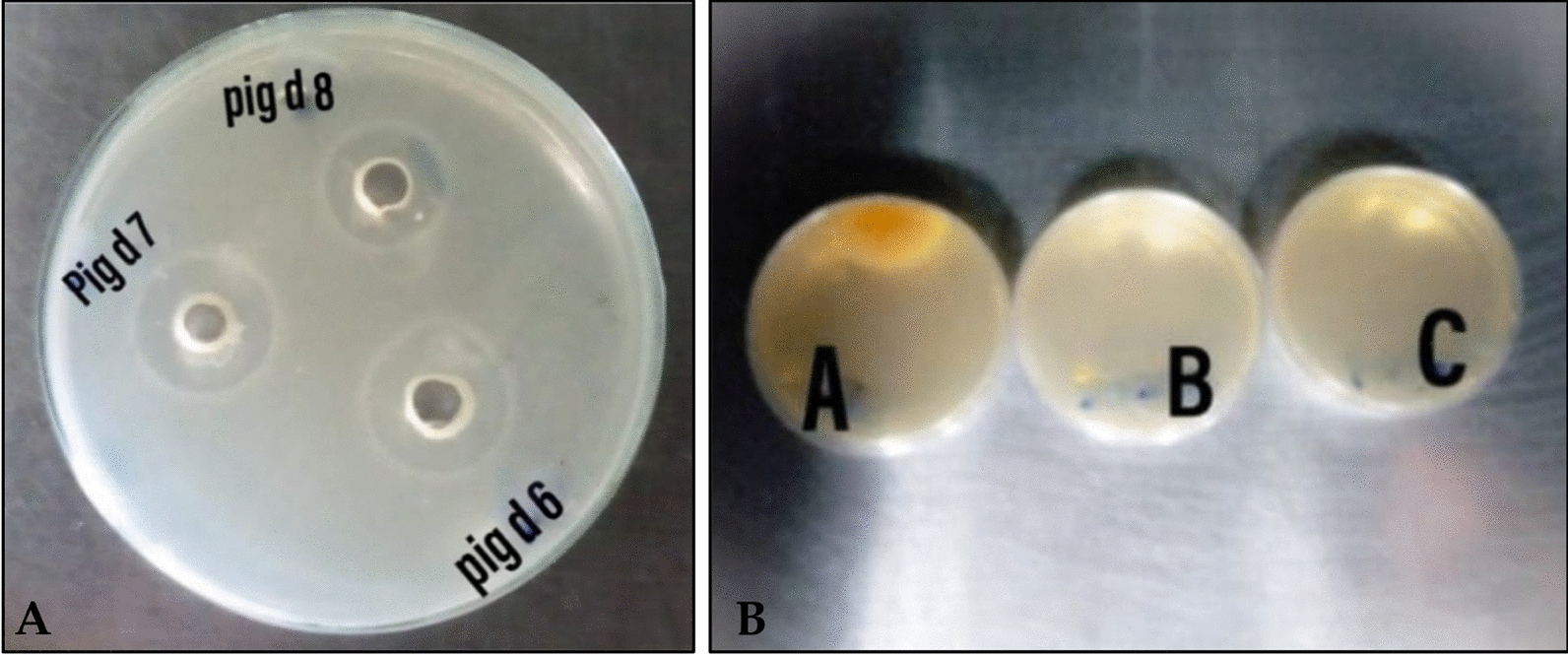
**A** Antibacterial activity of the *Streptomyces tunisiensis* W4MT573222 pigment against *Enterococcus faecalis* ATCC29212. **B** Antifungal activity of *Streptomyces tunisiensis* W4MT573222pigment against *Fusarium solani* ATCC10557, 50 µL (**B**), 100 µL (**C**) and control (**A**). Pig refers to pigment; d refers to day

The antibacterial effect of *Streptomyces tunisiensis*W4MT573222 pigment was tested against some pathogenic bacteria namely:* S. aureus*25923,* E. coli* ATCC 19404,* V. fluvialis, V. damsela*, and* E.faecalis*ATCC29212 using agar well diffusion method at 37 °C for 24 h incubation period. The result revealed that the extracted pigment showed an antibacterial effect against the tested bacterial pathogens at a concentration of 100 µL from the stock solution at a concentration of 2.5 mg/mL. Results revealed that the largest inhibition zone of 21 mm was noticed against* E. faecalis* ATCC 29212, followed by 18, 17, 16, and 15 mm inhibition zone against* V.damsela*, *E.coli*ATCC19404, *V. fluvialis*, and *S. aureus* 25923, respectively as shown in Table 2. Figure 6A showed the antibacterial activity of the *Streptomyces tunisiensis* W4MT573222 pigment against *Enterococcus faecalis* ATCC29212 which was choosen because it is highly distributed in marine habitats via sewage water and, as a result, it causes some human infectious diseases.

**Table 2:** Antibacterial activity of *Streptomyces tunisiensis* W4MT573222 pigment against some pathogenic bacteria

Bacterial pathogens					
Pig.conc (2.5 mg/mL)	*S. aureus* ATCC25923	*E. coli *ATCC19404	*V. fluvialis*	*V. damsela*	*E. faecalis *ATCC29212

Inhibition zone diameter(mm)					
(100 µL)	15	17	16	18	21

### Antifungal activity of the *Streptomyces tunisiensis* W4MT573222 pigment.

The antifungal effect of *Streptomyces tunisiensis* W4MT573222 pigment was tested against some pathogenic fungi namely: *Fusarium solani* ATCC10557*, Rhizoctonia solani* ATCC6599, and* Candida albicans* to determine its antifungal activity. After incubation for 72 h at 37 °C it was found that the extracted pigment showed an efficient antifungal activity against all the tested fungal pathogens. The results revealed that there was no growth of the fungal pathogens at the two different concentrations of the *Streptomyces tunisiensis* W4MT573222 pigment (50 µL and 100 µL) that were used from the stock solution at a concentration of 2.5 mg/mL Fig. 6B.

### The anti-oxidant capacity of *Streptomyces tunisiensis* W4MT573222 pigment

The extracted pigment of *Streptomyces tunisiensis* W4MT573222 exhibited an antioxidant capacity at 2500 µg/mL concentration, which is equal to 37 µmol/g butylhydroxytoluene (BHT) at the same concentration using phosphomolybdenum reagent. A spectrophotometric method has been developed for the quantitative determination of antioxidant capacity. The assay is based on the reduction of Mo(VI) to Mo(V) by the sample analyte and the subsequent formation of a green phosphate/Mo(V) complex at acidic pH [17].

### Antiviral activity of the extracted pigment

**Fig. 7:**
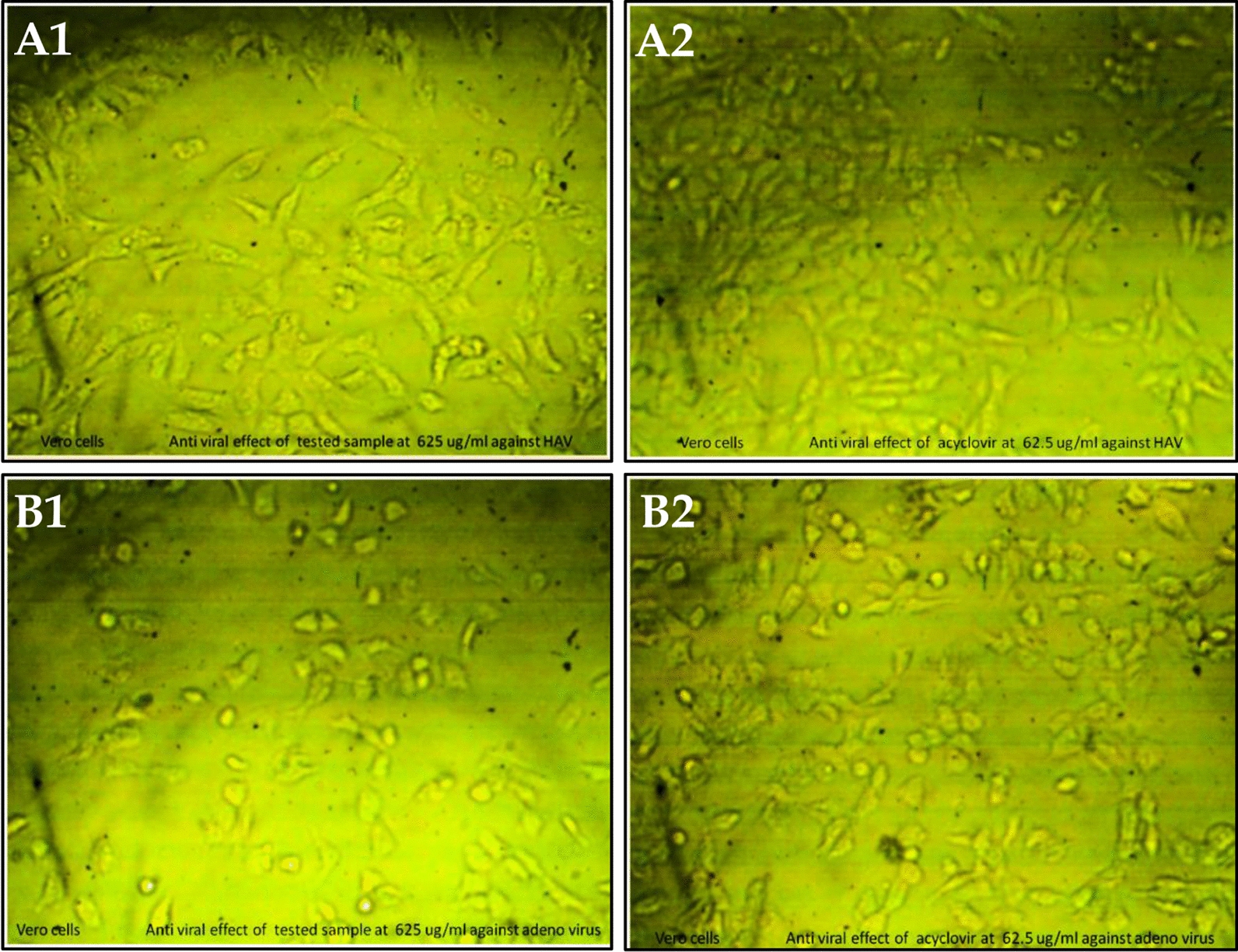
**A1** Antiviral effect of *Streptomyces tunisiensis*W4MT573222 pigment at a concentration of 625 µg/mL against HAV(all the actions done through HAV have been decreased by 78%). **A2** Antiviral effect of acyclovir at a concentration of 62.5 µg/mL against HAV. **B1** Antiviral effect of *Streptomyces tunisiensis*W4MT573222 pigment at a concentration of 625 µg/mL against Adenovirus (all the actions done through Adenovirus have been decreased by 13%). **B2** Antiviral effect of acyclovir at a concentration of 62.5 µg/mL against Adenvirus

**Fig. 8:**
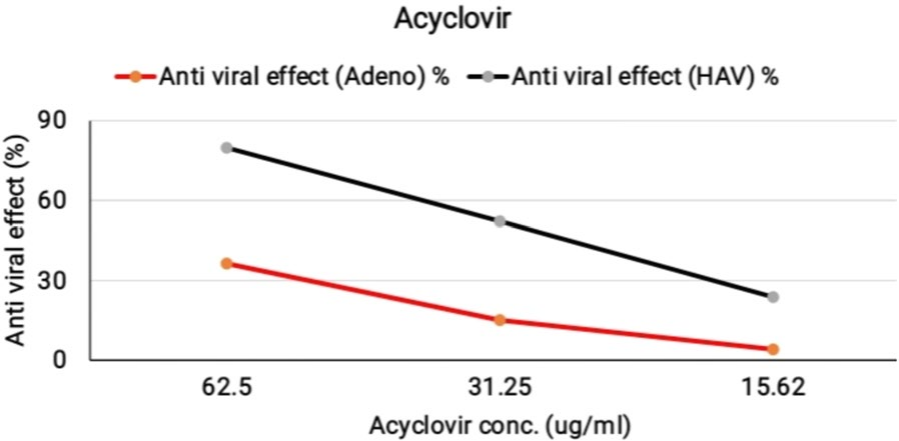
Antiviral effects of Acyclovir at different concentrations against HAV and Adenovirus

The cytotoxicity of the *Streptomyces tunisiensis* W4MT573222pigment was examined on human normal and cancer cell lines. The cytotoxic effects of different concentrations of the extracted pigment and acyclovir (as antiviral control) against normal human kidney epithelial cells (Vero cells) were evaluated. The cytotoxic effects of extracted pigment and acyclovir (the used antiviral control Fig. 7A2, B2 and Fig. 8 were evaluated by measuring the O.D. (optical density of cells) at 560 nm after treatment with extracted pigment and acyclovir individually. The maximum non-toxic concentration [MNTC] of the extracted pigment was 625 µ/mL while the maximum non-toxic concentration [MNTC] of acyclovir was 62.5 µg/mL.

**Fig. 9:**
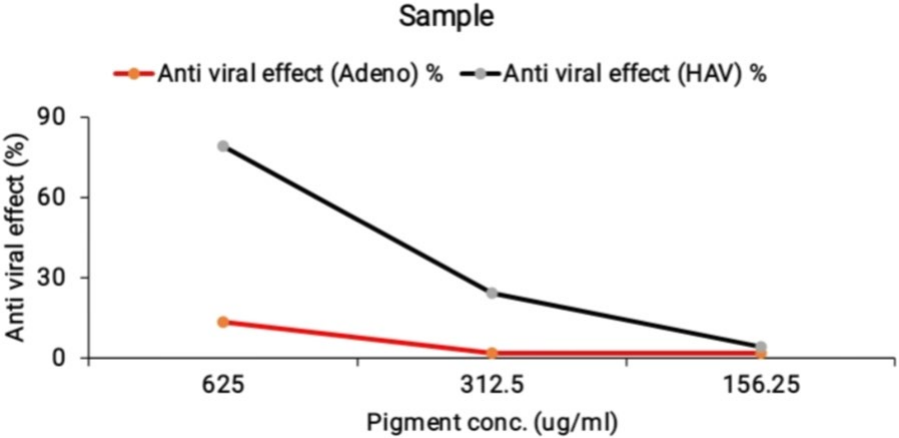
Antiviral effects of *Streptomyces tunisiensis* W4MT573222 pigment at different concentrations against HAV and Adenovirus

The antiviral effects of different concentrations of the tested pigment and acyclovir against the Hepatitis A virus and Adenovirus were evaluated using MTT functional assay. The antiviral effect of different concentrations of the tested pigment and acyclovir (as antiviral control) was evaluated by measuring the OD at 560 nm after treatment with the tested pigment and acyclovir, the optical density should be directly correlated with the cell quantity. The results indicated that the pigment has antiviral activity at different concentrations against HAV Fig. 7A1 and Fig. 9; however, it has a weak antiviral effect at different concentrations against Adenovirus Fig. 7B1 and Fig. 9**.**

### Anticancer activity of the extracted pigment

**Fig. 10:**
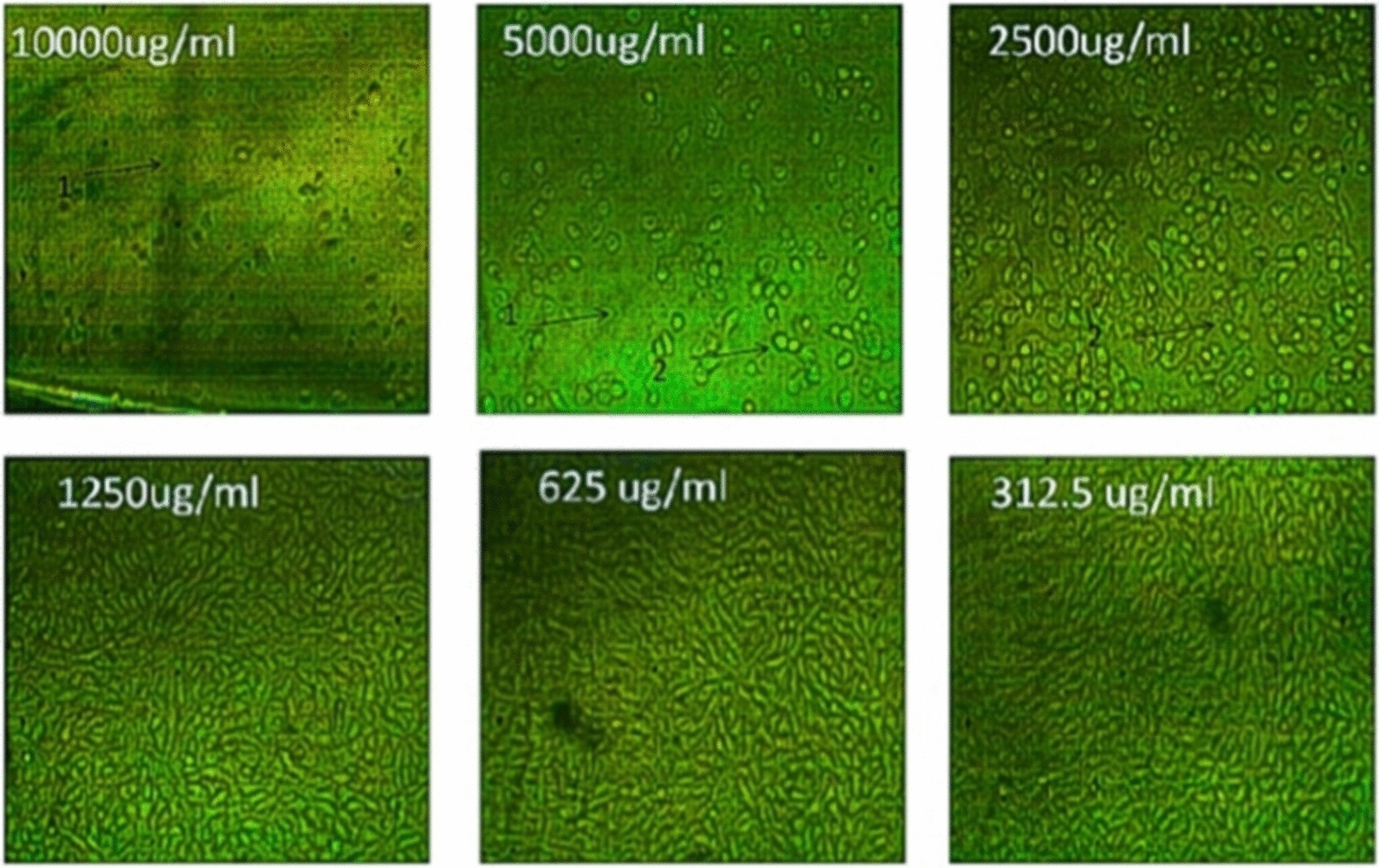
Effect of *Streptomyces tunisiensis* W4MT573222 pigment against HepG2 cells showed complete loss of the monolayer sheet of cells at concentration 10,000 µg, while at concentration 5000 µg showed partial loss of the sheet (1) and rounding cells without granulation (2) appearance of normal sheet was observed at concentration 2500 µg with same rounding cells (3) while at the rest of concentration the normal sheet was observed

**Fig. 11:**
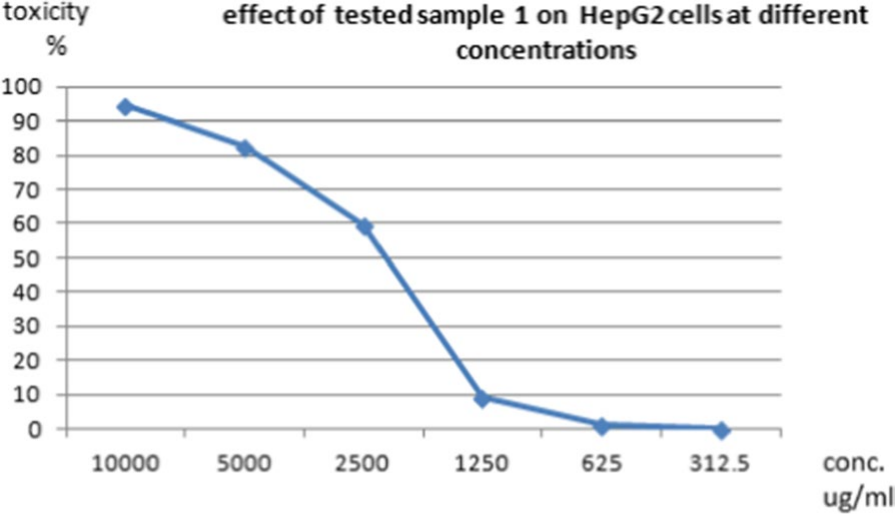
Effect of *Streptomyces tunisiensis* W4MT573222 pigment on HepG2 cells at different concentrations

**Fig. 12:**
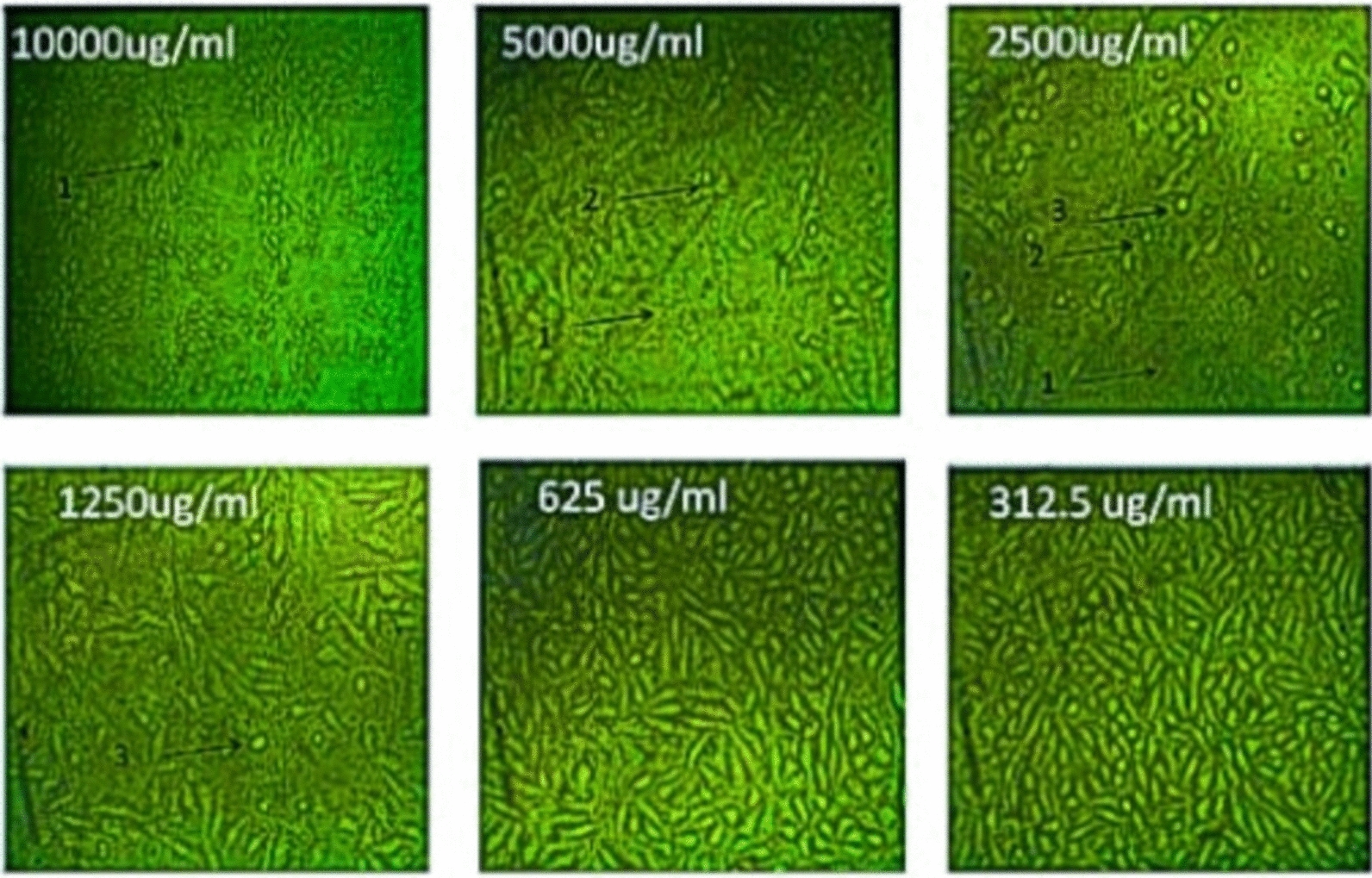
Effect of *Streptomyces tunisiensis* W4MT573222 pigment against A549 cells showed complete loss of the monolayer sheet of cells at concentration 10,000 µg, while at concentration 5000ug showed partial loss of the sheet (1) and shrinkage of cells (2) appearance of normal sheet was observed at concentration 2500 µg with same rounding cells (3) and shrinkage cells and partial loss of the monolayer sheet, same rounded cells were appeared at concentration of 1250 µg/mL, while at the rest of concentration the normal sheet was observed

**Fig. 13:**
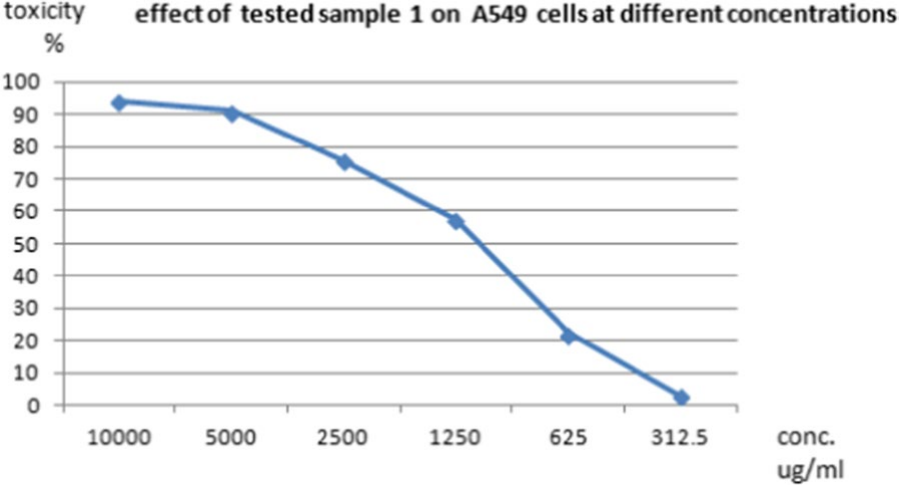
Effect of *Streptomyces tunisiensis* W4MT573222 pigment against A549 cells at different concentrations

**Fig. 14:**
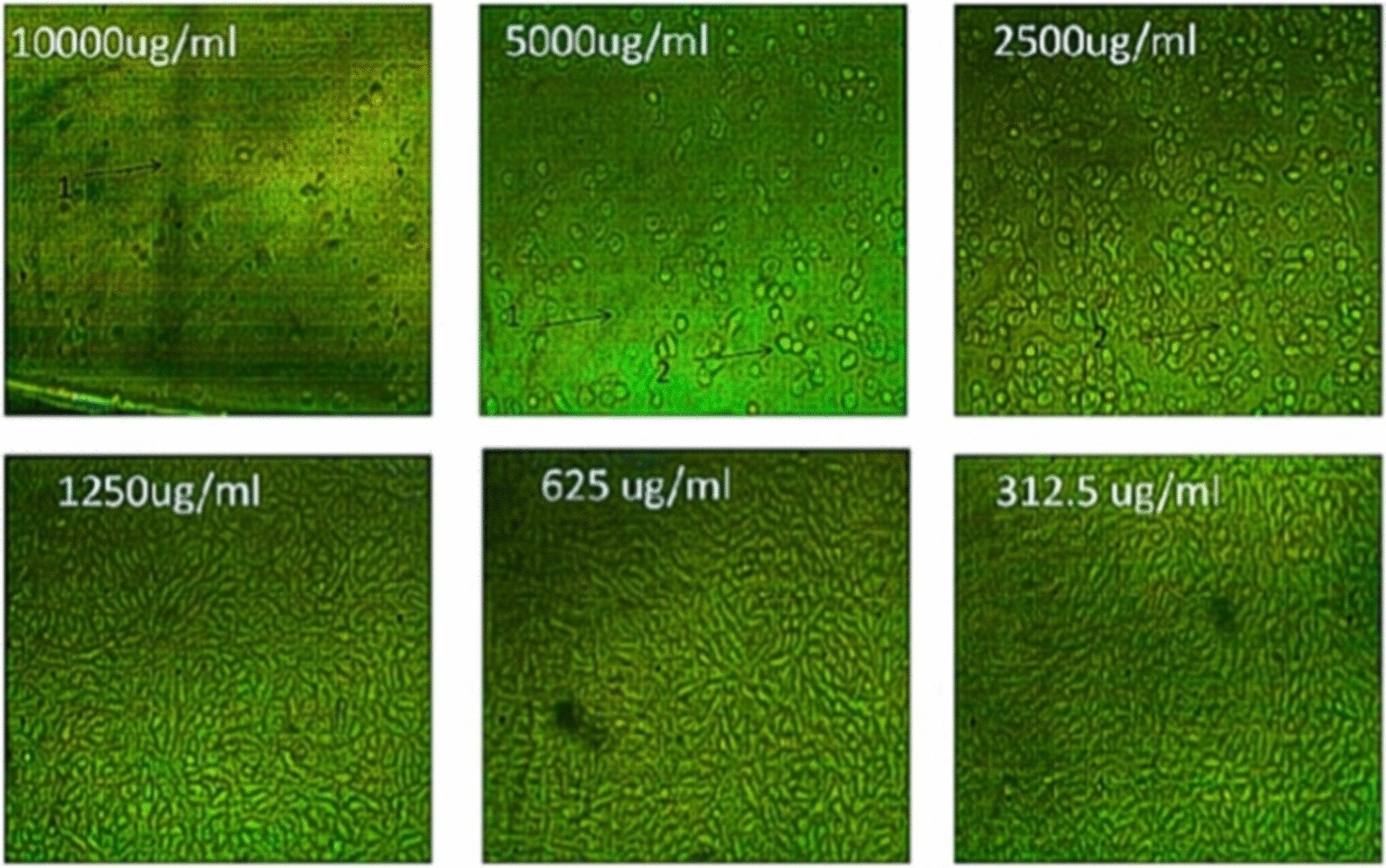
Effect of *Streptomyces tunisiensis*W4MT573222pigment against PANC1 cells showed complete loss of the monolayer sheet of cells at concentration 10,000 µg, while at concentration 5000 µg showed partial loss of the sheet (1) and shrinkage of cells (2) appearance of normal sheet was observed at concentration 2500 µg without any cytopathic effect, while at the rest of concentration the normal sheet was observed

**Fig. 15:**
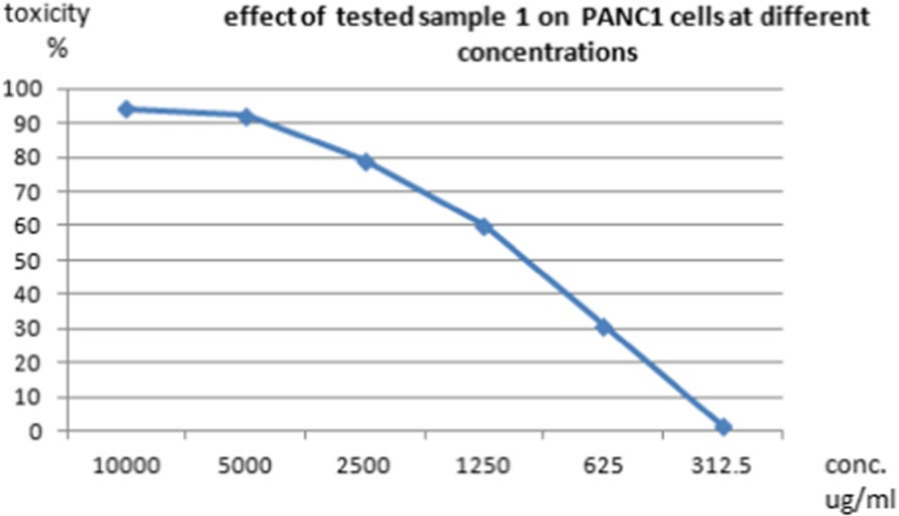
Effect of *Streptomyces tunisiensis* W4MT573222 pigment against PAN1 cells at different concentrations

The cytotoxic effects of different concentrations of the tested pigment and Doxorubicin (as a positive control) on normal cell line as well as on different cancer cell lines such as HepG-2 (liver cancer cell line ATCC HB-8065), A549 (lung cancer cell line ATCC CCL-185), and PAN1 (pancreas cancer cell line ATCC CRL-1469) were evaluated using MTT functional assay. The results revealed that the treatment of normal and cancer cell lines with different concentrations of the tested pigment showed cytotoxic effects on the tested cell lines. The high IC_50_ values are indicators of the safety of the extract toward normal cells. This means that the highest IC_50_ values of pigment proved its highest safety, while, the low IC_50_ value is an indicator of high anticancer activity. These results indicated that the IC_50_ of the extracted pigment proved its anticancer potential against HepG-2 (liver cancer cell line) as shown in Figs. 10 and 11, A549 (lung cancer cell line) as shown in Figs. 12 and 13**,** and PAN1 (pancreas cancer cell line) as shown in Figs. 14 and 15. The IC_50_ values of the extracted pigment were 2277 µg/mL (2.2 mg/mL), 1114 µg/mL (1.1 mg/mL), and 1043 µg/mL (1 mg/mL) against HepG-2 (liver cancer cell line), A549 (lung cancer cell line) and PAN1 (pancreas cancer cell line) respectively, while IC_50_ of extracted pigment against Vero cells (normal cells) was 2014 µg/mL (2 mg/mL) which prove the high safety of extracted pigment. However, The IC_50_ values of the Doxorubicin were 19.32 µg/mL, 23.52 µg/mL, and 22.86 µg/mL against HepG-2 (liver cancer cell line), A549 (lung cancer cell line) and PAN1 (pancreas cancer cell line) respectively.

### The combination between commercial antibiotics and the tested pigment

The extracted pigment was combined with 9 commercial antibiotics namely: Ciprofloxacin (CIP), Levofloxacin (LEV), Ceftriaxone (CRO), Amoxicillin (AX), Cefuroxime (CXM), Gentamicin (CN), Cefadroxil (CFR), Amoxicillin-Clavulinic Acid (AMC), and Cefotaxime (CTX), then were tested against Gram-negative bacterium *Enterococcus faecalis* using disc diffusion bioassay one at a time.

**Fig. 16:**
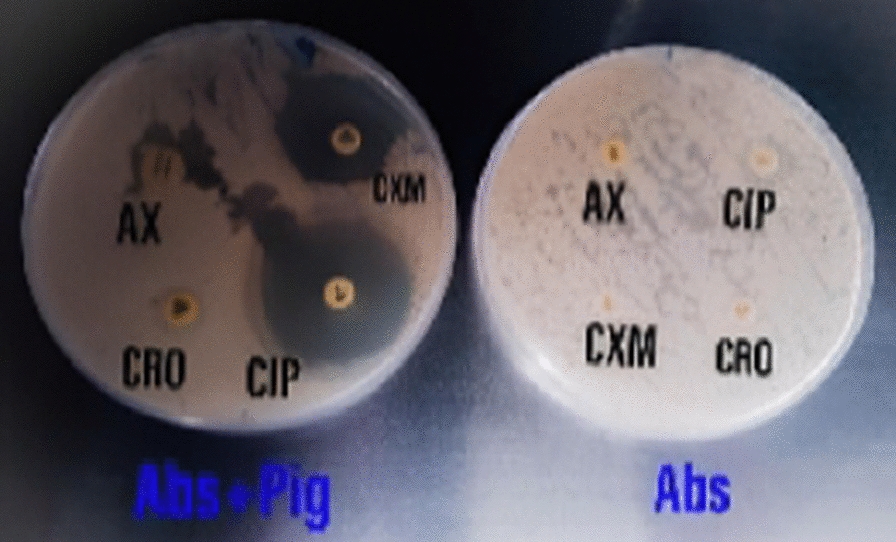
Effect of the combined action of some commercial antibiotics and* Streptomyces tunisiensis* W4MT573222 extracted pigment against *Enterococcus faecalis* ATCC29212

Table 3 revealed that the extracted pigment showed an antibacterial effect against *E. faecalis* ATCC29212 with an inhibition zone diameter of 21 mm, while in combination with different antibiotics reported variable results. The combined action of the pigment with Levofloxacin (LEV, 5) was antagonistic. On the other hand, the combined action of the pigment with cefuroxime (CXM, 30) was synergistic; similarly, Ciprofloxacin (CIP, 5) showed a synergistic effect. The combined action of some commercial antibiotics and* Streptomyces tunisiensis* W4MT573222 extracted pigment against *Enterococcus faecalis* ATCC29212 was shown in Fig. 16.

**Table 3:** Combined action of antibiotics and the extracted pigment

Antibiotics (µg)	Inhibition zone diameters (mm)				
	Single drug	Drug interaction (calculated)	Drug interaction (observed)	synergistic/antagonistic index	Drug interaction interpretation
Pigment only	21				
Ciprofloxacin (CIP, 5)	14	(14 + 21) 35	39	1.11	Synergistic effect
Levofloxacin (LEV, 5)	30	51	40	0.78	Antagonistic effect
Ceftriaxone (CRO, 30)	0.0	21	8	0.38	Antagonistic effect
Amoxicillin (AX, 25 IU)	0.0	21	12	0.57	Antagonistic effect
Cefuroxime (CXM, 30)	0.0	21	25	1.19	Synergistic effect
Gentamicin (CN, 10)	18	39	28	0.72	Antagonistic effect
Cefadroxil (CFR, 30)	18	39	18	0.46	Antagonistic effect
Amoxicillin-Clavulinic Acid (AMC, 30)	15	36	18	0.50	Antagonistic effect
Cefotaxime (CTX, 30)	7	28	14	0.50	Antagonistic effect

All the antibiotics were mixed with the tested pigment (250 µg/100µL)

## Discussion

One of the main threats to healthcare in the world is antimicrobial resistance, as stated by WHO. To counter this issue, new drugs with action against antibiotic-resistant microorganisms are urgently required to be tested. Natural compounds isolated from marine sources may play a significant role in discovering new antimicrobial agents. Due to the special condition of the marine environment, actinomycetes are particularly desirable due to their high ability to produce bioactive compounds [18].

Rasool and Hemalatha [19] revealed that the marine actinomycetes constitute a group of less exploited marine microbes, being the richest source of novel, and essential compounds. Actinomycetes in general and marine actinomycetes in particular have been a source of numerous life-saving antibiotics such as chloramphenicol, tetracycline, erythromycin, and other essential antibiotics. Until 1974 this genus *Streptomyces* was the only source available for antibiotic development. Compared to their terrestrial correlatives, marine actinomycetes have a different ability to synthesize products due to the different surroundings around them, such as the synthesis of enzymes, bioactive secondary metabolites, and antibiotics. Marine actinomycetes have served as a source of novel antibacterial, antifungal, and anticancer compounds. Therefore they play a significant role in the sciences of biology.

Ramesh and Mathivanant [20] found that, out of 208 isolates, 6 produced diffusible pigment on starch casein agar, and 58 isolates produced exopolysaccharides. The pigments and exopolysaccharides (EPS) production could act as protective mechanisms for actinomycetes to survive in the hostile marine environment.

FTIR technique is used to obtain an infrared spectrum, emission, and photoconductivity of a solid, liquid, or gas. FTIR working in the mid-infrared region (4000 to 400 cm^−1^) is an effective method for fat, oils, and palm carotene quantitative analysis [21]. The bands of the characteristic functional groups (CH_3_, CH_2_, C=C, C=O, OH, etc.) can be assigned when possible. Some special functional groups such as C=C=C, ‘cross epoxides’, etc., which cannot be easily identified by giving full form (1H-NMR) methods, can be detected in the FTIR. In this study, FTIR revealed the presence of OH and N–H (amine), and that agreed with Chakraborty et al. [22] in their study on bio pigment from actinobacteria. Also C–Br and C–I (alkyl halides), C–O–C, and C=C (alkene) were detected and that coincided with Hegazy et al. [23] in their study on pigment produced from marine *Natrialba* sp. In addition, Tandale et al [24] revealed the presence of the following functional groups OH, NH, CH, CN, and COOH in their research on lip balm production from pigment-producing actinomycetes.

Advances in Raman microscopy allow non-destructive technical studies of paper-based historical manuscripts to be performed to identify pigments and inks from texts and decorations. A particular benefit of Raman spectroscopy is the ability to focus on individual particles, thus improving the specificity of analysis. The discrimination between the paper and the writing and drawing media proves to be less difficult when the data are provided with minimum interference from either binders or neighbouring pigments, due to the high spatial and spectral resolution of the instrumentation [25].

The result revealed that the tested pigment has an antibacterial effect against some bacterial pathogens (*S. aureus* 25923, *E. coli* ATCC19404 *V. fluvialis*, *V. damsela*, and *E. faecalis* ATCC29212). It was found that the tested pigment has antifungal activity against all the tested fungal pathogens (*Fusariumsolani* ATCC10557, *Rhizoctoniasolani* ATCC6599, and *Candida albicans*). The obtained result was an agreement with that recorded by Amal et al. [26], who found that dark brown pigment produced by *Streptomyces virginiae* showed activity against Gram-negative and positive bacteria (*Pseudomonas aeruoginosa* and *S.aureus*) as well as fungi (*F. oxysporium*, *Botrytis allii*, *Diplodiaoryzae*, *A. flavus*, and *A. niger*) On the other hand, Kheiralla et al. [27] clarified that the antibacterial activity of the produced pigments against both Gram-positive (*B. cereus* and *S. aureus*) and Gram-negative (*E. coli* and *S. typhi*) strains was evaluated in their study and the results indicated that the pigment did not have any activity against the tested microbes. In addition, in the case of antifungal activity of the produced pigment(s), these pigments didn't have any activity against tested fungi.

Concerning Adenovirus treatment, the tested pigment showed only 13.3% inhibition, whereas acyclovir achieved 35.9% inhibition, i.e. the tested pigment was ineffective in the treatment process. This disagreed with Strand et al. [28], who reported that the anti-adenoviral activity of marine actinobacteria extract was characterized and substantial differences in the cytotoxic potential between the butenolide analogs were observed. The treatment of hepatic virus (HAV) with the extracted pigment and acyclovir gave similar values (78.5 and 79.1) respectively. Cytotoxic activity of crude extract of *Streptomyces tunisiensis* W4MT573222 SA-13 against the Vero cell line has been reported at various concentrations. The crude extract with IC-50 200 μg mL displayed moderate toxicity. The strong association between antimicrobial activity and cytotoxicity was evident. With increasing concentration, both antimicrobial activity and toxicity increase [29].

Bhat et al. [30] illustrated that the anticancer activity was assessed by employing 96-well plate MTT assay and flow cytometry-assisted apoptosis on human prostate adenocarcinoma (PC-3) cells. The primary indication of nanoparticles synthesis was given by a colour change from slight yellow to brown. While, Parmar and Singh [31] revealed that the toxicity effect on two cell lines HDF (Human dermal fibroblast) and U-87 (Human Glial cell) cell lines were procured from National Centre for Cell Sciences (NCCS) were studied on different concentrations (31.25, 62.50, 150, 250, 500, 1000 and 2000 µg/mL). It is found that cell inhibition is increasing as the dose of pigmented compound increases on both cell lines. The cytotoxic effect of the pigmented compound showed CTC50 > 1000 μg/mL which indicates that pigmented compound has very low cytotoxicity or is mildly toxic against both cell lines. The study confirmed that the natural pigment has a very less cytotoxic effect and is probably used in food and pharmaceutical industries as a natural colorant agent. The compound is more effective against cancer cell lines as compared to normal cell lines, these results agreed with our results as IC50 of pigment extract proved its anticancer potential against HepG-2, A549, and PAN1. The IC50 of pigment extract against HepG-2 was 2277 µg/mL (2.2 mg/mL), the IC50 of pigment extract against A549 was 1114 µg/mL (1.1 mg/mL), and the IC50 of pigment extract against PAN1 was 1043 µg/mL (1 mg/mL), while IC50 of pigment extract against Vero cells (normal cells) was 2014 µg/mL (2 mg/mL) which prove the high safety of pigment extract toward normal cells.

## Conclusion

Misuse and overuse of antibiotics for maintaining human health have been major contributing factors to the rapid emergence and spread of antibiotic-resistant bacteria, which pose a serious threat to global public health. *Streptomyces tunisiensis*W4MT573222 pigment showed antimicrobial effects against different pathogens (bacterial and fungal pathogens). The results revealed that the* Streptomyces tunisiensis* W4MT573222 pigment had a synergistic effect when combined with some commercial antibiotics. The pigment might be used to improve the efficiency of infectious disease treatments when combined with commercial antibiotics.* Streptomyces tunisiensis*W4MT573222 pigment had an antioxidant effect. Results revealed that the *Streptomyces tunisiensis*W4MT573222 pigment had an antiviral effect against the Hepatitis A virus. *Streptomyces tunisiensis*W4MT573222 pigment showed an anticancer effect against some tumor cell lines. The cytotoxic effect of *Streptomyces tunisiensis*W4MT573222 pigment proved the safety of the pigment extract towards normal cells. The results illustrated that *Streptomyces tunisiensis* W4MT573222 pigment has significant antimicrobial and antitumor effects against potential pathogenic microbes and tumor cell lines respectively. Therefore in the future, it is recommended to be used as a professional candidate in different medical applications.

## Materials and methods

### Isolation and culture medium composition

Samples were collected from sediments of the Abu-Qir coast of the Mediterranean Sea in Alexandria, Egypt. Sediment samples (ten samples) were collected from a depth of 5–20 cm below the surface of each location using piston corer in clean plastic bags. Samples were immediately transported to the laboratory in an ice box for bacteriological analysis which was always completed within 24 h. The isolation medium composition was contained(g/l): Starch, 10 g; Casein 0.30 g; KNO_3_, 2 g; K_2_HPO_4_, 2 g; MgSO_4_.7H_2_O, 0.05 g; FeSO_4_.7H_2_O, 0.01 g; CaCO_3_,0.02 g; agar, 18 g [32]. The culture medium was prepared using seawater. The pH was adjusted to 7, and the medium was sterilized by autoclaving at 120 °C for 20 min.

The primary screening of actinomycetes was performed as following: sediment samples without previous dilutions were taken, 10 g from each sample were added to 30 ml sterilized sea water, shacked for 20 min, then subjected to thermal treatment (by heating in a water bath at 50 °C for 1 h) to reduce the number of unicellular bacteria in favor of actinomycetes. Starch casein agar plates were inoculated with 1 ml of the treated samples. The medium was supplemented with 75 and 25 μg ml^−1^ of filter-sterilized cycloheximide and nystatin respectively to minimize fungal contamination [33]. Actinomycetes were recognized by their characteristic tough leathery colonies, branched vegetative mycelia, aerial mycelia and spore formation. Actinomycetes colonies observed from each sample were isolated by repeated transfer on starch casin agar medium for growth until pure strains were obtained as judged by colony morphology.

The actinomycetes isolates were screened for the production of antimicrobial agents (such as pigments). This refers to the potential of the actinomycetes isolates to produce those unique pigments. An actinomycete coded as W4 was isolated and further studies were completed. (Note: The pigment was extracted after 6, 7, and 8 days of the growth of the *Streptomyces tunisiensis*W4MT573222 to optimize the growth conditions for maximum pigment production).

### Molecular characterization

The genomic DNA of isolateW4 was extracted using the Gene Jet genomic DNA purification (Fermentas) kit, and the gene coding for 16SrRNA was partially amplified using Maxima Hot Start PCR Master Mix (Fermentas) and universal primers. The product of the PCR was analyzed on 1% agarose gel stained with ethidium bromide. Finally, isolateW4 was identified by sequencing the PCR-amplified 16S rRNA gene with 1500 bp, then the BLAST program was used to assess the similarity. The 16S rRNA of isolate W4 was submitted to GeneBank under accession number (MT573222). Then, the multiple sequence alignments were used to construct a phylogenetic tree using BioEdit Sequence Alignment Editor Program. (Sigma Scientific Services Co, 2013).

### Microscopic examination

For scanning electron microscopic examination of the selected actinobacterium, it was grown on starch casein broth medium for 7 days at 35 ± 2 °C. The culture was fixed in glutaraldehyde (2.5%, v/v), washed with water, and post-fixed in osmium tetroxide (1%, w/v) for 1 h. The sample was washed twice with water and dehydrated in ascending ethanol (30%, 50%, 70%, 90%, and 100%), and finally coated in gold and examined at 15–20 kV in JEOL JSM 5400 LV, scanning electron microscope, at the electron microscope unit of Alexandria University.

### Pigment extraction

The most potent selected strain was grown on Starch casein agar (SCA) at 37 °C for 7 days under shaking conditions (120 rpm). At the end of the incubation period, the culture broth was centrifuged at 10.000 rpm, for 20 min at 4 ℃. The supernatant was preserved at 4 ℃ (to extract the small part of diffusible pigment later) and 50 mL of distilled water was added to the pellets and then kept at 4 °C overnight. A mixture of acetone-methanol (7:3 v/v) containing butylhydroxytoluene (BHT) (0.1% as an antioxidant) was added to the pellets. Successive extractions were carried out until both solvents and cells were colorless and then centrifuged at 10.000 rpm, for 20 min at 4 ℃. The solvent was evaporated at 45 ℃ overnight; the pigments were dissolved in 50 mL acetone containing 0.1% BHT. The colored extracts were analyzed by scanning the absorbance in the wavelength region of 200-700 nm using the Perkin Elimer UV/VIS Lambda EZ 201, USA Spectrophotometer, where *λ*_*max*_was found at 300 nm.

### Identification of the extracted pigment

*UV absorption analysis*: UV–Vis spectrum was determined by UV–Vis spectrophotometer (Model: PerkinElmer-650). The wavelength at which the pigment has its strongest photon absorption was determined to detect λ_max_ of the tested pigment.

*Fourier transform infrared (FTIR) spectroscopy analysis*: the biological functional groups present in the acetone-methanol extract of pigment were determined by FTIR analysis, based on the peak values of the infrared spectrum [6]. The molecular structure of the dried extracted pigment was identified using a Peak Find-Memory-27 spectrophotometer. A mixture of approximately 1 mg of the tested compound and 300 mg of pure dry potassium bromide (KBr) was pressed into discs. The measurements obtained infrared spectra between 400 and 4000 cm ^− 1^ at the central lab, Scientific Researches and Technological Applications City, Borg El-Arab, Egypt**.**

*Raman spectroscopy*: a Raman Senterra instrument with a multi-wavelength capability operating at 785 nm with a power of 50 mW and a wide range of 400–4000 cm^ − 1^ was used to measure the effect of the excitation wavelength on the pigment spectrum. A laser irradiated an object in an optical microscope (laser spot = 2 μm), and the scattered light from the sample was collected by the optics of the microscope passing through holographic filters, a pinhole, and a monochromator to be detected by a charge-coupled device (CCD) at the central lab, Scientific Researches and Technological Applications City, Borg El-Arab, Egypt**.**

*EDX analysis*: energy Dispersive X-ray (EDX) analysis was carried out at the electron microscope unit, faculty of science, Alexandria University, Egypt, to identify the elemental composition of the extracted pigment using JSM-IT 200 instrument.

### GC–MS analysis

This analysis was performed according to a previously reported method [34] using A Trace 1300 GC Ultra/Mass Spectrophotometer ISQ QD (Thermo Scientific) instrument at the High Institute of Public Health-Alexandria University, Egypt. A 5 TG-5MS Zebron capillary column with dimensions of (length 30 m × 0.25 mm ID, 0.25 µm film thickness; Thermo) was used, and helium was used as the carrier gas at an average velocity of 39 cm/s. The column Temperature was increased from 180 to 200 °C for 2 min (5 °C/min), 200-240 ºC for 2 min (5 °C/min), and 240–280 °C (5 °C/min) for 4 min. The MS instrument was operated at 70 eV. The constituents were identified by a comparison of their mass spectral data with those of standard compounds from the National Institute of Standards and Technology (NIST) Spectral Library.

### Antibacterial activity

#### Agar well diffusion assay

Müeller-Hinton agar** (MHA)** plates were prepared and inoculated with the bacterial pathogens *S. aureus* ATCC25923*, E. coli* ATCC19404,* V. fluvialis*, *V. damsela*,* E .faecalis* ATCC29212, *P. aeruginosa* ATCC9027, *B. cereus*, *B. subtillus* ATCC6633, and* E. coli* ATCC8739. After solidifying, wells were punched out using 0.7 cm cork-borer. The actinobacterium was grown on starch casein broth medium for 7 days at 37 °C and pH7. At the end of the incubation time, the culture was poured into a falcon tube then the falcon tube was subjected to centrifugation at 5000 rpm for 30 min then the cell-free extract was poured into (50 mL) falcon tube, 100 μL of the extracted pigment was pipetted into each well and left for one hour in refrigerator for compound diffusion. All plates were incubated at 37 °C for 24 h. After the incubation period, the diameter of the clear zone around each well (Y) and the diameter of the well (X) were linearly measured in millimetres (mm). The absolute activity unit (AU) of each crude extract was calculated according to the following equation:

AU = Y^2^/X^2^. Since (Y) is the radius of the clear zone around each well; (X) is the radius of the well itself [35].

### Antifungal activity

The pigment of *Streptomyces tunisiensis* was assessed for its ability to produce antifungal agents to inhibit the fungal indicators. The fungal indicators used in the current investigation were; *Rhizoctonia solani* ATCC6599, *Fusarium solani*ATCC10557, and *Candida albicans*. The fungal biomass was spot-inoculated on the fungal media but the Candida albicans was inoculated to nutrient agar medium containing the tested pigment. Control fungal plates without pigment were used to measure the decrease in fungal growth that indicates the fungal activity of the product under investigation [17].

### Antioxidant capacity

The pigment (2.5 mg) was dissolved in 1 mL dimethyl sulfoxide, to reach a final concentration of 2500 µg/mL for the tested compound. The antioxidant activity of the pigment solution (200 µL) was investigated using phosphomolybdenum reagent (32 mM sodium phosphate, 4 mM ammonium molybdate, and 0.6 M sulfuric acid) according to Sikkandar et al. [36]. Finally, the absorbance was measured at 695 nm, and antioxidant capacity is expressed as standard butylhydroxytoluene equivalents (BHT).

 Antiviral activity using MTT assay (3-(4,5-dimethylthiazol-2-yl)-2,5-diphenyltetrazolium bromide).

The MTT assay is a colorimetric assay for assessing cell metabolic activity. NAD(P) H-dependent cellular oxidoreductase enzymes under defined conditions reflect the number of viable cells present. These enzymes are capable of reducing the tetrazolium dye MTT 3-(4,5-dimethylthiazol-2-yl)-2,5-diphenyltetrazolium bromide to its insoluble formazan, which has a purple color [37]. The viruses were purchased from Vacsera company [38].

- The antiviral effects of different concentrations of the tested pigment and acyclovir against the Hepatitis A virus and Adenovirus were evaluated using MTT functional assay. Plate 10,000 cells in 200ul media per well in a 96-well plate. Leave 3 wells empty for blank controls.
- Incubate (37 °C, 5% CO_2_) overnight to allow the cells to attach to the wells.
- Incubate equal volume (1:1 v/v) of non-lethal dilution of the tested sample and the virus suspension for one hour.
- Add 100 µL from viral/sample suspension (HAVor Adenovirus). Place on a shaking table, 150 rpm for 5 min.
- Incubate (37 °C, 5% CO_2_) for 1- day to allow the virus to take effect.
- Make 2 mL or more of MTT solution per 96-well plate at 5 mg/mL in PBS.
- Add 20 μL MTT solution to each well. Place on a shaking table, 150 rpm for 5 min, to thoroughly mix the MTT into the media.
- Incubate (37 °C, 5% CO_2_) for 1–5 h to allow the MTT to be metabolized.
- Dump off the media. (dry the plate on paper towels to remove residue if necessary.
- Resuspend formazan (MTT metabolic product) in 200 µL DMSO. Place on a shaking table, 150 rpm for 5 min, to thoroughly mix the formazan into the solvent.
- Read optical density at 560 nm and subtract background at 620 nm. Optical density should be directly correlated with cell quantity [38].

 Anticancer activity using MTT assay (3-(4,5-dimethylthiazol-2-yl)-2,5-diphenyltetrazolium bromide).the cytotoxic effects of different concentrations of the tested pigment on normal cell line as well as on different cancer cell lines such as HepG-2 (liver cancer cell line), A549 (lung cancer cell line), and PAN1 (pancreas cancer cell line) were evaluated using MTT functional assay. The cancer cell lines were purchased from the cell bank and have the ATCC number as follow: HepG2 (HB8065), A549 (CCL-185), PAN1 (CRL-1469) [38].

Determination of sample cytotoxicity on cells (MTT protocol).

- The 96-well tissue culture plate was inoculated with 1 × 0^5^ cells/mL (100 µL/well of HepG2, A549, or PAN1 cells) and incubated at 37 °C for 24 h to develop a complete monolayer sheet.
- The growth medium was decanted from 96 well microtiter plates after a confluent sheet of cells was formed, and the cell monolayer was washed twice with wash media.
- Two-fold dilutions of the tested sample were made in RPMI medium with 2% serum (maintenance medium).
- 0.1 mL of each dilution was tested in different wells leaving 3 wells as control, receiving only maintenance medium.
- The plate was incubated at 37 °C and examined. Cells were checked for any physical signs of toxicity, e.g. partial or complete loss of the monolayer, rounding, shrinkage, or cell granulation.
- MTT solution was prepared (5 mg/mL in PBS) (BIO BASIC CANADA INC).
- 20 µL MTT solution was added to each well. Place on a shaking table, 150 rpm for 5 min, to thoroughly mix the MTT into the media.
- Incubate (37 °C, 5% CO_2_) for 1–5 h to allow the MTT to be metabolized.
- Dump off the media. (dry the plate on paper towels to remove residue if necessary.
- Resuspend formazan (MTT metabolic product) in 200 µL DMSO. Place on a shaking table, 150 rpm for 5 min, to thoroughly mix the formazan into the solvent.
- Read optical density at 560 nm and subtract background at 620 nm. Optical density should be directly correlated with cell quantity [38].

### The combined action of the tested pigment and some antibiotics

The antibacterial activity of pigment under test was examined against the selected pathogen* (Enterococcus faecalis* ATCC29212) Because *E.* *faecalis* is highly distributed in marine habitats via sewage water, as a result, it causes some human infectious diseases.

A standard inoculum of 1.5 × 10^6^ CFU/mL equivalent to 0.5 McFarland was prepared (A550 ≤ 1) and 25 μL was swabbed over the surface of the MHA plate. Antibacterial activity was carried out using the disc diffusion method; discs of antibiotics were saturated with 100 μL of pigment extract (2.5 mg/1 mL) which was dissolved in DMSO, then placed on the surface of the inoculated MHA plates [39]. The plates were kept at 4 °C for 1 h for compound diffusion and then incubated for 24 h at 35 ± 2 °C. At the end of the incubation period, the inhibition zone diameters were recorded in millimeters and the results were the mean of three trials [40].

The results of antibiotics were added to the results of the tested pigment against Gram-negative bacteria (calculated data). Observed data divided by calculated data to demonstraste if the activity was synergistic, antagonistic, or additive effect depending on Synergistic/Antagonestic index calculation. The results are summarized as follows:

$$\mathrm{Synergistic}/\mathrm{antagonistic index}= \frac{\mathrm{Observed value}}{\mathrm{Calculated value}}$$

Synergistic/Antagonistic index > 1→Synergistic effect.

Synergistic/Antagonistic index < 1→Antagonistic effect.

Synergistic/Antagonistic index = 1→Additive effect.

## Data Availability

All data produced during this study are included in this published article.
